# Concrete with a High Content of End-of-Life Tire Materials for Flexural Strengthening of Reinforced Concrete Structures

**DOI:** 10.3390/ma15176150

**Published:** 2022-09-05

**Authors:** Thomaida Polydorou, Nicholas Kyriakides, Andreas Lampropoulos, Kyriacos Neocleous, Renos Votsis, Ourania Tsioulou, Kypros Pilakoutas, Diofantos G. Hadjimitsis

**Affiliations:** 1 Department of Civil Engineering and Geomatics, Cyprus University of Technology, Limassol 3036, Cyprus; 2 ERATOSTHENES Centre of Excellence, Limassol 3012, Cyprus; 3 School of Architecture, Technology and Engineering, University of Brighton, Brighton BN2 0JY, UK; 4 Department of Civil and Structural Engineering, The University of Sheffield, Sheffield S10 2TN, UK

**Keywords:** rubberized concrete, recycled steel fibers, rubber aggregate, End-of-life tire materials, strengthening

## Abstract

This research investigates the performance of Steel Fiber Reinforced Rubberized Concrete (SFRRC) that incorporates high volumes of End-of-life tire materials, (i.e., both rubber particles and recycled tire steel fibers) in strengthening existing reinforced concrete (RC) beams. The mechanical and durability properties were determined for an environmentally friendly SFRRC mixture that incorporates a large volume (60% by volume aggregate replacement) of rubber particles and is solely reinforced by recycled tire steel fibers. The material was assessed experimentally under flexural, compressive and impact loading, and thus results led to the development of a numerical model using the Finite Element Method. Furthermore, a numerical study on full-scale structural members was conducted, focusing on conventional RC beams strengthened with SFRRC layers. This research presents the first study where SFRRC is examined for structural strengthening of existing RC beams, aiming to enable the use of such novel materials in structural applications. The results were compared to respective results of beams strengthened with conventional RC layers. The study reveals that incorporation of End-of-life tire materials in concrete not only serves the purpose of recycling End-of-life tire products, but can also contribute to unique properties such as energy dissipation not attained by conventional concrete and therefore leading to superior performance as flexural strengthening material. It was found that by incorporating 60% by volume rubber particles in combination with recycled steel fibers, it increased the damping ratio of concrete by 75.4%. Furthermore, SFRRC was proven effective in enhancing the energy dissipation of existing structural members.

## 1. Introduction

Replacement of concrete aggregates by recycled rubber particles promotes sustainability and circular economy [1] and it particularly contributes to the elimination of End-of-life tires, an environmental and health issue recognized worldwide. Previous research [2] has identified the promising behavior of rubberized concrete, highlighting the fact that the inclusion of rubber particles as aggregate replacement can increase the energy absorption capacity of concrete significantly [3].

Even though researchers have advised against the use of high volumes of rubber particles due to the considerable compressive strength reduction, the use of larger quantities of rubber particles enhances particular concrete properties [4,5] that can improve the material behavior under impact and earthquake loading. The material flexural strength can be enhanced through the provision of adequate steel fiber reinforcement. Incorporation of two types of End-of-life tire materials (i.e., recycled tire steel fibers and recycled rubber particles) not only serves the purpose of recycling but can also increase the energy absorption of concrete [6], therefore making the material capable of absorbing energy conveyed to a structure during earthquake ground motion.

The application of novel fiber reinforced cementitious materials has been effectively used for the structural strengthening of existing Reinforced Concrete (RC) members including bridges [7] and beams [8,9,10]. Additional Steel Fiber Reinforced Concrete (SFRC) layers have been used with and without the presence of steel reinforcing bars [9,10], and the results have shown that the use of SFRC layers, even without steel bars, can significantly enhance the stiffness of structural elements, while the addition of steel reinforcing bars is essential for further enhancement of the ultimate load bearing capacity.

The use of materials such as SFRC provides significant benefits in terms of mechanical performance and durability, although traditional SFRC mixtures incorporate a large amount of manufactured steel fibers and are therefore costly and energy intensive. Hence, this research aims to suggest an alternative of using recycled steel fibers retrieved from End-of-life tires. Recycled tire steel fibers have been proven effective as concrete reinforcement, indicating equal or superior performance compared to manufactured steel fibers [11,12]. The mechanical properties of concrete mixtures incorporating recycled tire steel fibers and rubber particles at various contents and particle sizes has been a subject of many research studies [13,14,15,16,17,18], but the effect of using such materials to strengthen conventional RC beams, especially when including a high volume of rubber particles, has not been investigated previously.

Only one study has investigated the performance of polyvinyl alcohol (PVA) fiber reinforced rubberized cementitious composites in strengthening conventional RC beams thus far, indicating the promising potential of rubberized cementitious composites in strengthening and repairing RC structural members [19]. Thus, this research aims to evaluate for the first time the effectiveness in strengthening existing RC beams of concrete by incorporating a large volume of rubber particles (60% by volume aggregate replacement) and recycled steel fibers, both retrieved from End-of-life tires.

This study evaluates the mechanical and durability properties of environmentally friendly Steel Fiber Reinforced Rubberized Concrete (SFRRC). Experimental assessment included tests under static and cyclic compressive loading, flexural bending, impact loading as well as durability tests under chloride exposure and freeze–thaw cycles. In addition, numerical analysis has been conducted based on the obtained experimental data, which further enabled comparison to relevant conventional strengthening techniques.

## 2. Materials and Methods

The experimental study focused on an SFRRC mix design optimized by previous research [20], where 60% (by volume) of the mineral aggregate was replaced by rubber particles recycled from End-of-life tires. To maintain the aggregate particle size distribution in the mix, fine aggregate was replaced by finely granulated rubber of equivalent particle size, while coarse aggregate was replaced by shredded rubber of equivalent particle size.

The cement used was an EN 197-1 [21] Portland cement type CEM I 52.5 N, obtained from Vassiliko, Cyprus. In addition, we used Pulverized Fly Ash (PFA) according to EN 450-1 [22], and Microsilica or Silica Fume (MS) conforming to Silica Fume Class 1 requirements of EN13263-1:2005+A1:2009 [23]. A liquid polycarboxilic polymer-based superplasticizer conforming to EN934-2:2009 [24], with a specified mass density at 1070 kg/m^3^ ± 30 kg/m^3^ was also used in this study.

Diabase coarse aggregate (4–20 mm) and a blend of diabase and limestone fine aggregate (0–4 mm) from local quarries were used in this mix, at a fine to coarse aggregate ratio of 1:1.22. In replacement to 60% by volume of mineral aggregate, recycled rubber particles at equivalent sizes were used, particularly a blend of particles obtained from tire recycling plants in Croatia, Cyprus and the UK. Since no material specifications were provided, the specific gravity of a representative sample of the rubber particle blend used in this study was evaluated by EN 1097-6 [25]. The determined specific gravity was 0.8, which was taken into consideration during the mix design process, to ensure accurate aggregate replacement by volume.

Cleaned and sorted Recycled Tire Steel Fibers (RTSF) obtained from Sheffield, U.K. were used in this study. The evaluated tensile strength and Young’s Modulus of the RTSF used in this study were provided by the distributor at 2560 ± 550 MPa and 200,000 MPa ± 0.5%, respectively [26].

The SFRRC mix constituents are listed in Table 1. Specimen preparation followed standard concrete mixing procedures as described by EN 206:2013+A1:2016 [27], cast in standard cubes and cylinders as well as prisms of 10 × 10 × 40 cm^3^, demolded 24 h after casting and cured in a water bath in standard laboratory conditions. Before testing, the specimens were taken out of the water and allowed to dry for 24 h. before hardened concrete testing commenced.

To compare the response of SFRRC to Plain Concrete (PC), three standard cube PC specimens were cast as well as 6 10 × 10 × 40 cm^3^ rectangular PC prisms. The plain concrete mix constituents are listed in Table 1.

### 2.1. Static Loading

#### 2.1.1. Loading under Compression

The compressive strength of both cubic and cylindrical SFRRC specimens, as well as cubic PC specimens, was evaluated as prescribed by EN 12390-3:2009/AC:2011 [28]. The axial compressive load was applied on the cylinders at a displacement rate of 0.3 mm/min and on the cubes at a rate of 0.4 MPa/s, using a standard Compressive Testing Machine of 3000 kN load capacity. In addition, the static modulus of elasticity of cylindrical SFRRC specimens in compression was determined as recommended by BS 1881-121 [29].

#### 2.1.2. Loading under Bending

**Figure 1:**
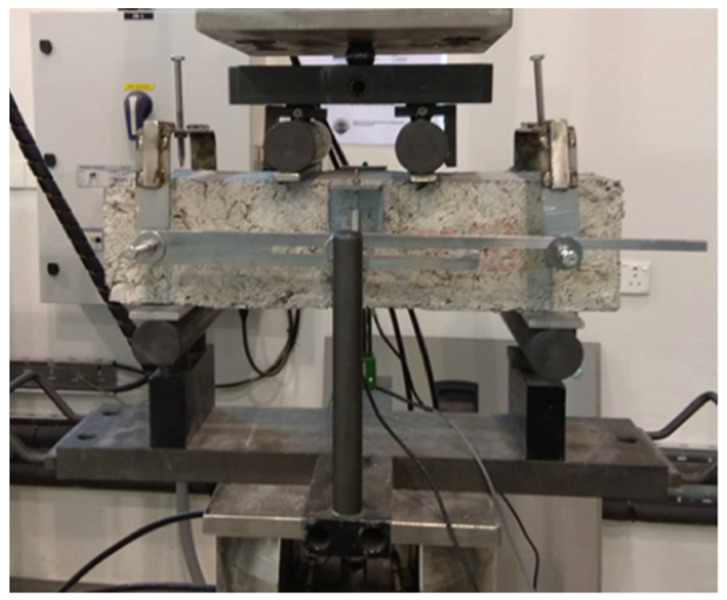
Four-point bending test setup.

Flexural strength was evaluated by loading prismatic specimens under four-point bending, using a state-of-the-art hydraulic water cooled tensile/compressive testing machine of 250 kN capacity. A custom-made yoke was used to mount two Linear Variable Difference Transducers (LVDTs), placed at midspan of each side of the prism as recommended by the JSCE-SF4 [30] method of tests for flexural strength and toughness of steel fiber reinforced concrete. Before initiating the test, a 12.5 mm long clip gauge was mounted on the bottom surface of the prism, to measure the crack mouth opening displacement (CMOD) on 5 mm wide, 15 mm deep notches, pre-sawn across the midsection of the prism. The bending test setup is shown in Figure 1.

Assessment of the post peak energy absorption of the material was conducted by studying the residual flexural strength (f_Ri_) values obtained at the predetermined crack mouth opening displacements (CMOD) of 0.5 mm, 1.5 mm, 2.5 mm, and 3.5 mm, as recommended by RILEM TC 162-TDF [31] and the EN 14651 standard [32]. To compare the response of SFRRC to plain concrete, three plain concrete rectangular prisms were also loaded under 4-point bending, using standard procedures and identical setup as for the SFRRC prisms, shown in Figure 1.

### 2.2. Cyclic Compressive Loading

**Figure 2:**
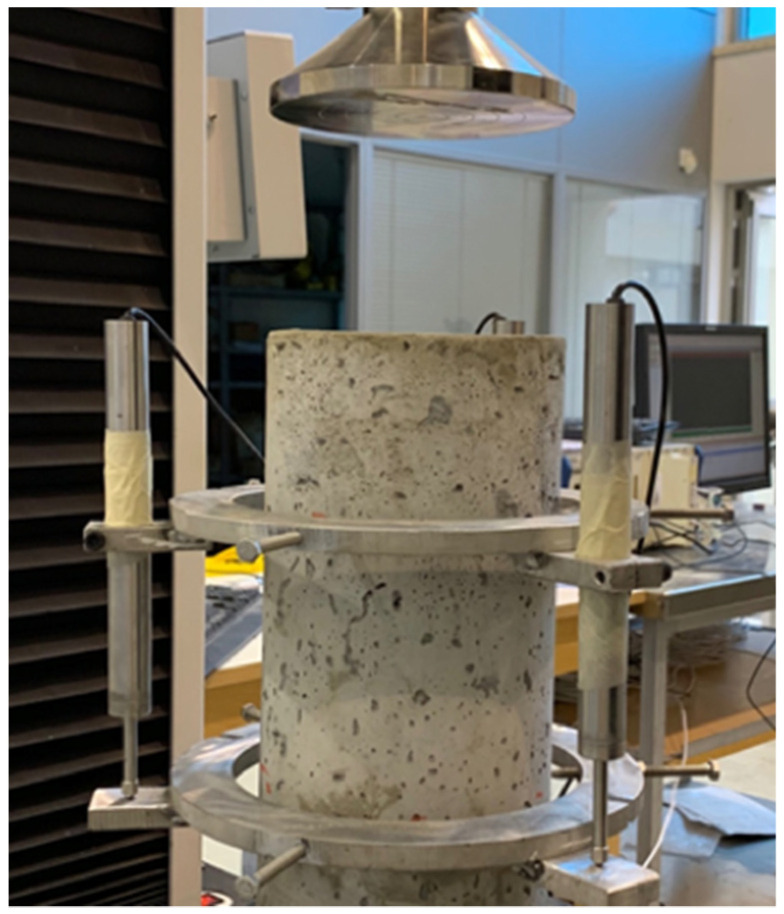
Cyclic compressive loading specimen setup.

The energy absorption of the material was also evaluated by loading cylindrical specimens (15 cm diameter) under cyclic compressive loading and using a universal load and displacement control testing machine. A custom-made yoke was used consisting of two circular brackets, each secured on the cylinder surface by two pins at 180-degree angle from each other and holding three LVDTs, arranged at 120-degree angles around the circumference of the specimen. The compressive loading setup is shown in Figure 2.

**Figure 3:**
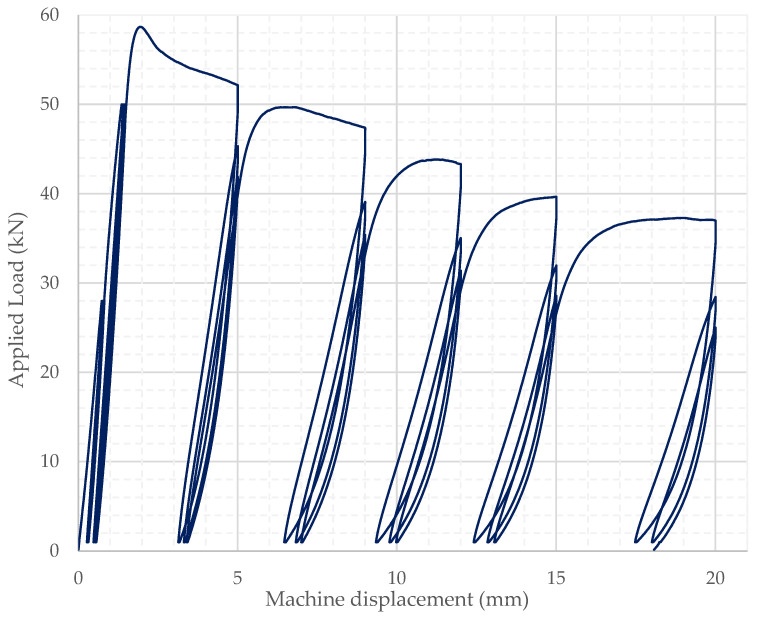
Cyclic loading command path.

Loading was applied by considering the average cylinder compressive strength from the respective cylinder batch and followed a specific loading command. All specimens followed identical loading commands, with the only variable being the maximum load reached. Testing was initially load controlled at a rate of 500 N/s up to a predetermined load limit of 28 kN, then followed by a 60 s pause before unloading down to 1 kN and reloading back to 28 kN twice; these cycles completed the first set of cyclic loading within the linear stress–strain behavior range. Loading continued without a pause at the same load-controlled rate up to 50 kN, before changing to displacement-controlled loading at a rate of 0.2 mm/min. The second set of loading cycles resumed at that rate by unloading down to 1 kN and loading back up to 50 kN, recurrently for a total of three cycles. Further on, specimens were loaded up to their capacity at the same displacement-controlled rate and, after that additional loading commenced to perform three cyclic loading and unloading rounds at each predetermined displacement of 5 mm, 9 mm, 12 mm, 15 mm and 20 mm, as indicated in the load-displacement plot shown in Figure 3. The displacement of the SFRRC Cylinder was determined based on the average displacement of the three mounted LVDTs.

### 2.3. Impact Loading

**Figure 4:**
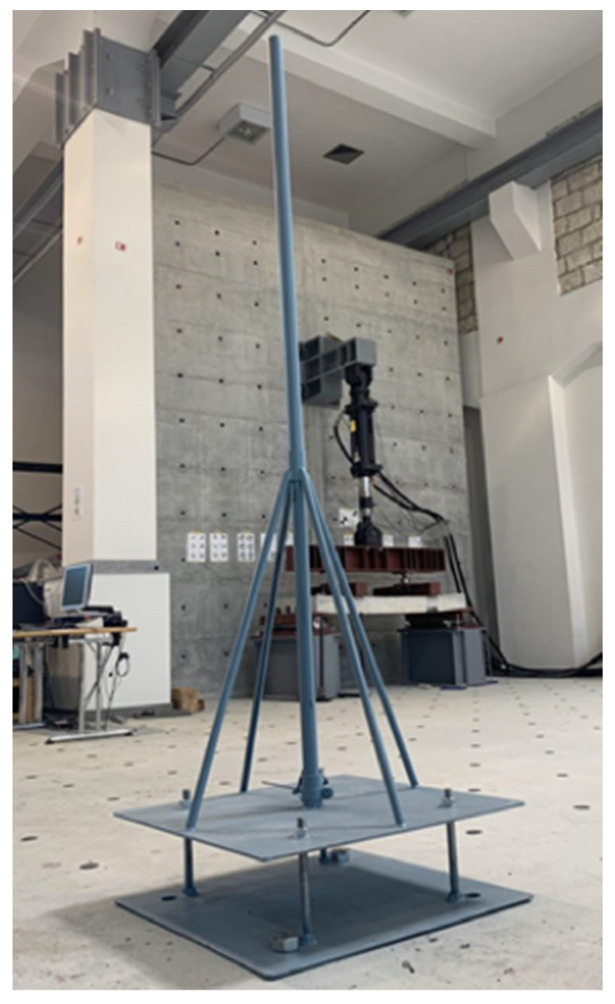
Impact test drop-weight frame.

The material response to impact loading was assessed by direct impact of a steel sphere that fell free from a known height through a cast-iron, cylindrical tube onto the top surface of rectangular 10 × 10 × 40 cm^3^ specimens. Six SFRRC and three PC specimens were experimentally assessed under impact loading in this study. A custom-made frame was specifically machined for this study and bolted to the laboratory’s strong floor to ensure stability, as shown in Figure 4; the design of this custom-made frame was based on the drop-weight impact test concept.

A stationary Kistler accelerometer with sensitivity of 1 V/g was used, mounted at the centroid of the impact surface of each prism, 150 mm from the point of impact. A Kyowa PCD-300B sensor interface and its respective DCS-100A recording software (Kyowa, Tokyo, Japan) were used for data acquisition. The sampling frequency was set to 1000 Hz to capture the first vibrational modes.

The scope of this test was to measure the damping ratio ζ of both SFRRC and PC, through the calculation of the decrease in magnitude of the vibration response at the mid span of the specimen.

### 2.4. Durability Testing

#### 2.4.1. Chloride Corrosion Exposure

Resistance to chloride corrosion was assessed by subjecting three sets of three rectangular, 10 × 10 × 40 cm^3^ prisms to wet–dry cycles. The specimens were exposed to chlorides by immersion into 3% NaCl under a schedule of 7 days in and 7 days out of solution repeatedly for 2-, 4- and 6-month periods. The mass of each prism was recorded prior to their first wet–dry cycle and upon conclusion of the respective set cycling period and compared to a fourth (control) set of prisms, which followed the same wet–dry cycling scheme but was immersed in clean water instead of NaCl solution.

#### 2.4.2. Freeze–Thaw Resistance

The freeze–thaw durability of the material was assessed by the mass scaling loss of four cubes (15 cm side), as recommended by the CEN/TS 12390-9:2016 [33] method. Thermocouples were embedded into the cubes during casting to enable specimen temperature monitoring through the freeze–thaw cycles. The cubes were weighed at 28 days maturity before being immersed in 3% NaCl solution in stainless steel containers and placed into a freeze–thaw chamber, programmed to apply continuous cycles of freezing and thawing by alternating between −15 °C and 20 °C. To determine mass loss, the cubes were removed from the chamber during thawing at the end of each 7-, 14-, 28-, 42- and 56-day cycle, and their surfaces were thoroughly brushed, which led to material detaching from the cubes due to scaling. The detached material was collected, oven dried at 105 °C for 24 h and then weighed to the nearest 0.1 g.

## 3. Experimental Results and Discussion

The paper examines the properties of a SFRRC mix design that includes a high content of End-of-life tire materials such as rubber particles and steel fibers under static and cyclic compressive loading, flexural bending, impact loading and durability testing. Local materials were used to recreate an already optimized SFRRC mix with 60% by volume of its mineral aggregate replaced by a blend of rubber particles with a specific gravity of 0.8 (as determined by EN 1097-6 [25]), as expected for a blend of rubber particles obtained from End-of-life tires. Mix development was achieved through aggregate replacement by volume and was based on the specific gravity of the rubber particle sample used. The experimental behavior of cubic, cylindrical and rectangular SFRRC specimens are discussed in the following subsections.

### 3.1. Static Loading

#### 3.1.1. Loading under Compression

The compressive strength of three cubic (15 cm side) and six cylindrical (15 cm diameter) SFRRC specimens were determined. An average 28-day cube compressive strength of 8.2 MPa was reached, whereas an average 28-day cylinder compressive strength of 3.5 MPa was obtained. The unexpectedly lower average compressive strength yielded by the cylindrical SFRRC specimens compared to their respective SFRRC cubic specimens is discussed as follows. To compare with PC, the compressive strength of three PC cubes (15 cm side) was also determined following standard procedures. The PC cubes reached an average 28-day cube compressive strength of 51.92 MPa, with a variance at 0.232.

The average static modulus of elasticity in compression was also determined for the high rubber content SFRRC mix by testing four cylinders (15 cm diameter), resulting in an average value of 3.263 GPa. The SFRRC static loading results summary is provided in Table 2.

As widely accepted, aggregate replacement by rubber particles leads to a significant reduction in concrete compressive strength. In this study, a compressive strength reduction of 84.2% was observed, expected due to the high percentage of the mix aggregate being replaced by rubber particles when comparing PC cubes to SFRRC cubes.

Focusing on the SFRRC compressive test results, where both cubes and cylinders were compared, a notable 134.29% higher compressive strength was obtained for SFRRC cubes compared to SFRRC cylinders from the same batch, much greater than the discrepancy expected due to their geometry. The mix consistency is one of the reasons the cylinder compressive strengths did not reach the values expected based on their respective cube strengths. It was observed that rubber particles tend to rise to the surface soon after casting, due to their lightweight nature. Therefore, slender cylinders end up inconsistent through their height and fail in the top 1/3 of the specimen where rubber is accumulated creating a larger, weak Interfacial Transition Zone (ITZ) that leads to premature rupturing. This explains the early failure of cylinders compared to cubes in compression, thus signifying that the extremely low compressive strengths yielded by the cylinders are not realistic and could have been much higher had the mix consistency been ensured.

The uneven distribution of rubber in the cylinder results in a failure zone in the top part of the cylindrical specimen, as described in Section 3.2. It is possible that the standard procedure for mixing and placing conventional concrete is not suitable for rubberized concrete. The cylinder samples yielded significantly lower compressive strengths than their respective cubic specimens due to uneven distribution of the rubber particles in the taller cylindrical specimens. It is recommended that alternative procedures for mixing and placing rubberized concrete are developed, specifically targeting the addition of rubber particles to the mix and their accumulation to the top part of the specimen during placement, providing cohesive mixes of uniform properties. A potential solution could be to use pre-treated rubber particles, using techniques proven [6,34] to enhance mechanical properties of the material, or even placing rubber particles separately from the rest of the SFRRC mix constituents.

#### 3.1.2. Loading under Bending

**Figure 5:**
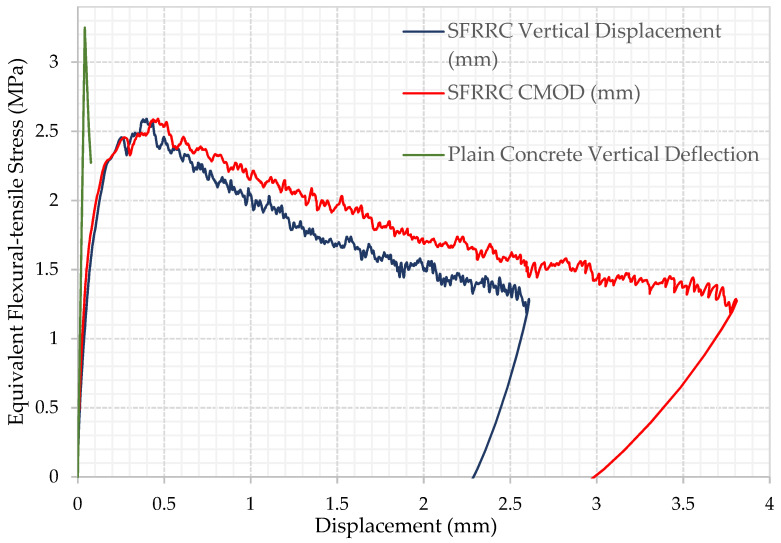
Equivalent Flexural-tensile stress vs. Displacement for Steel Fiber Reinforced Rubberized Concrete Specimen P5 and Plain Concrete Specimen PC1.

**Figure 6:**
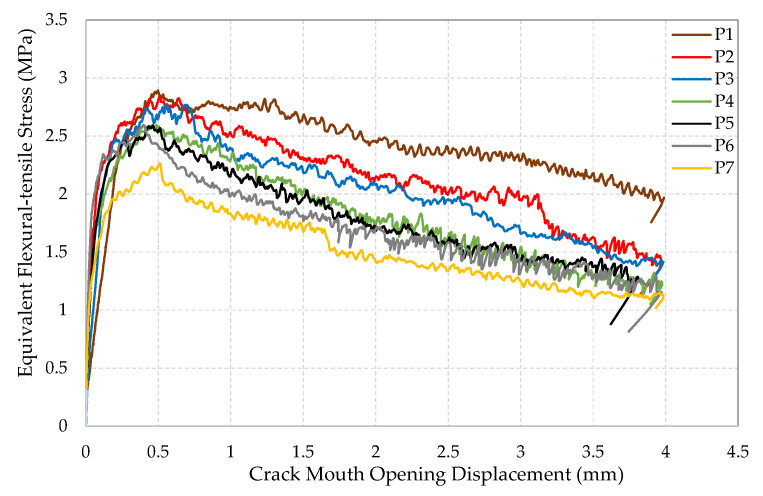
Equivalent Flexural-tensile stress vs. CMOD.

**Figure 7:**
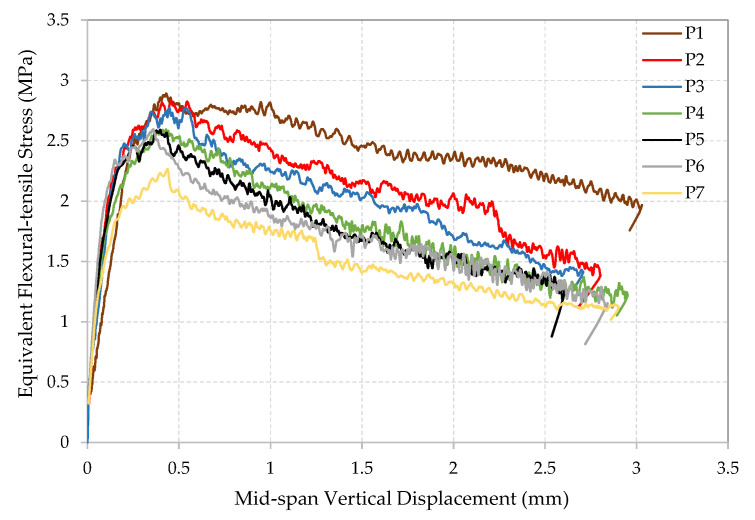
Equivalent Flexural-tensile stress vs. Mid-span Vertical Displacement.

The equivalent flexural-tensile stress versus crack mouth opening displacement (CMOD) and the average of the two LVDT readings (Vertical Displacement) were recorded for each of the seven 10 × 10 × 40 cm^3^ rectangular SFRRC prisms tested under 4-point bending, as shown in Figure 5, Figure 6 and Figure 7. All specimens tested under bending came from two batches of the same SFRRC mix. For comparative reasons, three PC rectangular specimens with dimensions of 10 × 10 × 40 cm^3^ were also tested under 4-point bending using the setup shown in Figure 1. The Equivalent Flexural-tensile Stress for PC specimen PC1 vs. Vertical Displacement is shown in Figure 5, along with the Equivalent Flexural-tensile Stress withstood by SFRRC specimen P5 vs. its Vertical Displacement and CMOD.

The residual flexural strength (f_Ri_) of each SFFRC prism was determined at the predetermined crack mouth opening displacements (CMOD) of 0.5 mm, 1.5 mm, 2.5 mm, and 3.5 mm, as recommended by RILEM [31]. The average flexural residual strength values and coefficients of variation obtained per CMOD are reported in Table 3. The limit of proportionality [32] of the prisms tested in this study was evaluated at an average value of 2.7 MPa.

Processing of the critical deflection values taken at points on the curves where the stress–strain behavior of the material is still linear during the static bending test rounds, in conjunction with the experimental modulus of elasticity (E) values obtained from cylinder compressive strength tests allowed the authors to determine the effective moment of inertia (I_eff_) of the specimens. The I_eff_ was calculated using Equation (1) [35]:

(1) $$\delta =k\frac{W\;L3}{E\;I_{\mathrm{eff}}}$$

using the experimentally determined E value, the experimental CMOD at midspan, determined at the maximum load sustained by each prism as δ, the theoretical value of k for simply supported beam setup, the total load (W) including the specimen self-weight and the measured length (L) of each prism.

The average effective moment of inertia (I_eff_) of the beam specimens tested under 4-point bending was determined at 1.5316 × 10^5^ mm^4^. This value was used to compare to the average value of specimen stiffness (k) obtained from the impact testing applied to beam specimens made of the same material mix and attaining the same geometrical characteristics.

The equivalent flexural-tensile stress versus crack mouth opening displacement (CMOD) plot shapes indicate the significant ability of the material to absorb energy, identified by the large area under the curve. In addition to the inclusion of the recycled tire steel fibers, the post peak energy absorption is attributed to the ability of the rubber particles to undergo large deformation in tension thus promoting high energy absorption. In contrast, PC does not attain nearly as much energy absorbing capability, as indicated in the PC Equivalent Flexural-tensile stress vs. Displacement plot included in Figure 5.

In addition to the graphs, the average SFRRC residual flexural strength values and the 43.6% reduction in residual flexural strength between the CMODs of 0.5 mm and 3.5 mm, as presented in Table 3, demonstrate that the SFRRC mix is highly ductile and attains exceptional post-cracking load carrying capacity. The SFRRC mix with a 60% by volume aggregate replaced by rubber particles has demonstrated high ductility and high post-cracking load carrying capacity, also indicated by high f_Ri_ values (Table 3), compared to the obtained limit of proportionality of 2.7 MPa.

The low rate of reduction in residual strength (f_Ri_) values observed (43.6% reduction between f_R1_ and f_R4_) also proves the capability of the SFRRC to attain post peak energy absorption. The deformability and toughness of the material is indicated not only by the visible large area under the stress–strain curve observed in the equivalent flexural-tensile stress vs. CMOD plots Figure 6, but also by the small reduction observed in the average flexural residual strength values (f_Ri_) in accordance with their corresponding and significant CMOD growth, as reported in Table 3.

The post cracking residual flexural-tensile strength of the SFRRC mix was classified by considering the provisions of the fib 2010 Model Code [36,37]. With an f_R1k_ at 2.25 MPa and f_R2k_ at 1.22 MPa, the material post-cracking residual strength is specified as class 2a, since the strength interval (f_R1k_) is between 2.0 and 2.5 MPa and the residual strength ratio ($\frac{\mathrm{fR}3k}{\mathrm{fR}1k}$) between 0.5 and 0.7 for class a, as defined by fib Model Code 2010 5.6-1; in addition, the characteristic limit of proportionality, f_LK_ [36] was calculated at 2.33 MPa, therefore a value of 0.2964 was obtained for the $\frac{\mathrm{fR}1k}{\mathrm{fLk}}$ ratio. Since both relationships of the fib Model Code 2010 [36] 5.6-2 (Equation (2)) and 5.6-3 (Equation (3)) are fulfilled, fiber reinforcement in this case could substitute (also partially) conventional reinforcement at ultimate limit states.

(2) $$\frac{\mathrm{fR}1k}{\mathrm{fLk}}>0.4$$

(3) $$\frac{\mathrm{fR}3k}{\mathrm{fR}1k}>0.5$$

### 3.2. Cyclic Compressive Loading

**Figure 8:**
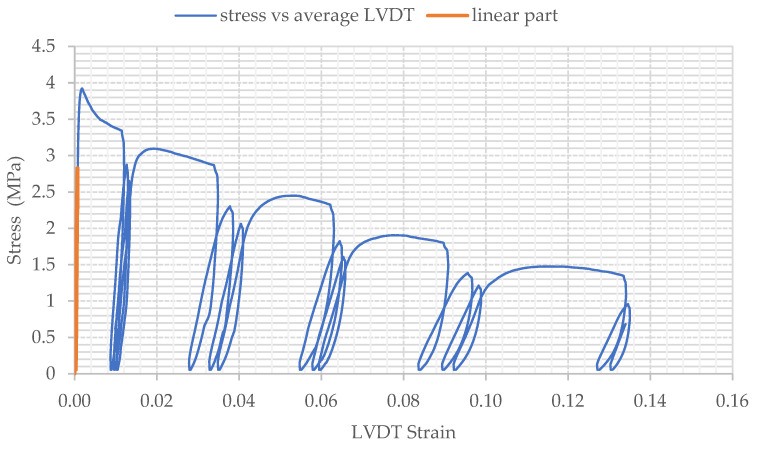
Stress vs. Average Strain Curve for Specimen B2 under Cyclic Compressive Loading.

Four standard SFFRC cylindrical specimens (15 cm diameter) were loaded under cyclic compressive loading to further confirm the energy absorption capabilities of the developed SFRRC mix with 60% by volume aggregate replaced by rubber particles. The stress–strain behavior of the cylinders tested in this study is represented by plotting the applied stress vs. average strain recorded by the 3 LVDTs mounted around the cylinder circumference, which were monitored throughout the application of cyclic loading and unloading rounds. The stress–strain plot obtained for cylinder B2 tested in this study is presented in Figure 8.

The cyclic compressive loading stress–strain curve shown in Figure 8 indicates the capacity of the material to withstand continuous cycles of axial compressive loading and unloading even post-peak.

**Figure 9:**
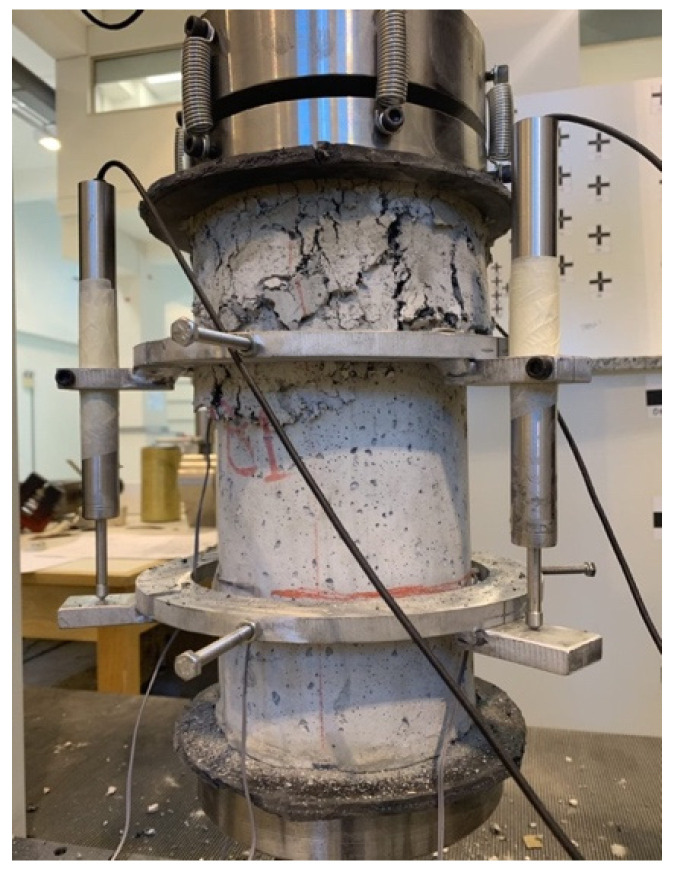
Cyclic Compressive Loading Specimen B1 at 20 mm Displacement.

The cylinder damage initiation and development of crack formation was visually observed during the cyclic loading and unloading program. The SFRRC mix with 60% aggregate replaced by rubber was able to reach an average ultimate strain of 0.023 before crack initiation. Figure 9 displays cylinder deformation at the end of the test, after completion of the final cyclic loading/unloading rounds at 20 mm machine displacement.

The average axial compressive strain at which significant cracking of the cylindrical specimens was observed was determined by visual inspection during the loading cycles and the results obtained by the LVDT measurements. As shown in Figure 9, the failure zone of specimen B1 is concentrated on the top part of the cylinder, indicating the inconsistency of material mix content through the cylinder specimen height. This explains the low compressive strength values obtained by the cylinders tested under static compressive loading in this study. In this case, the cylinder specimen tested had accumulation of rubber in the top 1/3 of the cylinder height.

The stress–strain behavior and shape of the curves shown in Figure 8 indicate the capacity of the SFRRC mix to absorb energy. The amount of energy absorbed by each specimen during the cyclic loading and unloading rounds is directly related to the area under their resulting stress–strain curves. The strain values before failure, corresponding to stress values that were no lower than 80% of the maximum or peak strength reached by each specimen, were considered as the ultimate value of strain reached by each specimen before crack initiation.

Visual inspection of the cylinders during the cyclic rounds of testing confirmed the material’s condition at the point of 80% peak strength. The strain values noted are relatively conservative, considering the number of loading and unloading cycles applied to the specimen prior to that.

### 3.3. Impact Loading

**Figure 10:**
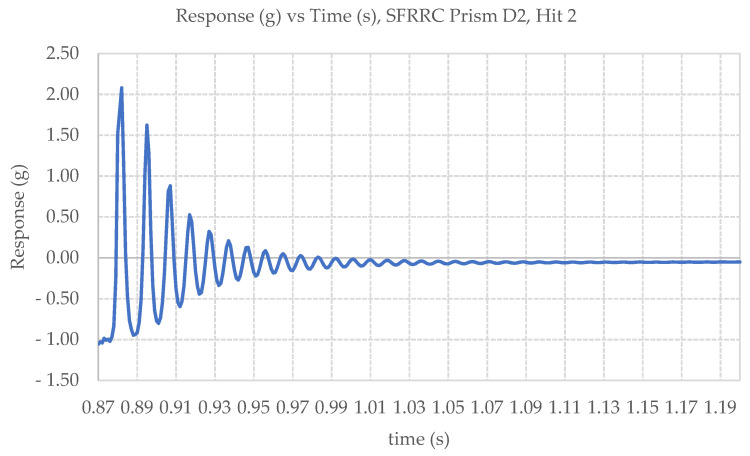
Impact Induced Oscillation Diagram for SFRRC Prism D2, Hit 2.

Impact loading was applied onto the top surface of 10 × 10 × 40 cm^3^ prismatic specimens. Three identical impact hits were performed on each specimen. The displacement response time history after impact loading was plotted for every hit and were further used to determine the damping characteristics for each of the tested specimens. Figure 10 presents an indicative oscillation diagram obtained for SFRRC specimen D2, under Hit 2, from this study.

The average damping ratio, ζ, and average fundamental frequency of oscillation, f, were calculated for the set of six prismatic SFRRC specimens tested in this study, taking into consideration the average values of ζ and f obtained by the three impact hits recorded per specimen. The damping ratios were calculated using values three peaks apart from each other, as indicated in Equation (4) [38]:

(4) $$\zeta =\frac{1}{2\pi \;j}\ln\frac{u\;i}{u\;j+i}$$

The fundamental frequency of oscillation, f, was determined in the frequency domain using Fast Fourier Transform (FFT). The average values of both ζ and f are listed in Table 4 along with their corresponding value of variance.

The damping ratio, ζ, and average fundamental frequency of oscillation, f, were calculated for PC specimens also, following identical procedures, for comparison purposes. The PC average damping ratio, ζ, was evaluated at 0.067 with a variance of 0.183, and the average frequency, f, for PC was determined at 52.00 Hz with a variance of 0.085, considering three impact hits on each of the three PC prisms tested in this study.

The average damping ratio (ζ = 0.118) of the SFRRC specimens tested in this study is 136% higher, relative to the widely accepted value for reinforced concrete (ζ = 0.05) [39], indicating superior energy absorbing capacity. In direct comparison to the impact performance of equivalent PC prisms tested under the same conditions during this study, there is a significant increase observed in the material damping ratio. Specifically, a 60% by volume replacement of aggregate by rubber particles led to a 75.4% increase in the value of ζ.

From the experimentally obtained frequency of oscillation, f, of the SFRRC specimens examined under impact loading during this study and reported in Table 4, the authors were able to obtain the stiffness, k, of each tested specimen, thus their moment of inertia, using the also experimentally obtained modulus of elasticity, E.

The effective moment of inertia (I = 2.9863 × 10^5^ mm^4^) of the SFRRC was calculated using the experimental parameters obtained from the impact test study. The stiffness (EI) of the specimen was calculated using the calculated value of circular frequency (ω) and the mass of the specimen was incorporated in the corresponding equation for the stiffness (K) calculation, accounting for the respective support and loading conditions. The derived I was found to be satisfactorily close to the effective moment of inertia, I_eff_ = 1.5316 × 10^5^ mm^4^, obtained using the experimental deflection of the specimen at specific loading points in the linear section of the load-deflection curves obtained in the static bending study. More specifically, the moment at mid span from four-point bending test and the modulus of elasticity obtained from compression load testing, were used to define I_eff_.

### 3.4. Durability Testing

#### 3.4.1. Chloride Corrosion Resistance

Four groups of prisms were examined under the chloride corrosion test program. One of the three groups, the control set, followed identical wet–dry cycling schedule as the other three groups, but was instead immersed in water with no chlorides present. The average mass loss and coefficients of variation per group of prisms tested in this study are reported in Table 5.

In terms of durability, chloride exposure mass loss values are insignificant, and the fact that the control set of specimens which underwent the cycles in plain water with no NaCl content has experienced similar mass loss compared to the specimen sets that were immersed in 3% NaCl solutions, proves that the developed SFRRC is not susceptible to corrosion when exposed to Chloride environments. The minor mass loss that occurred in all 4 sets of specimens is attributed to the loss of small pieces of material from the corners of prisms due to handling of the specimens through the duration of the study.

#### 3.4.2. Freeze–Thaw Resistance

**Figure 11:**
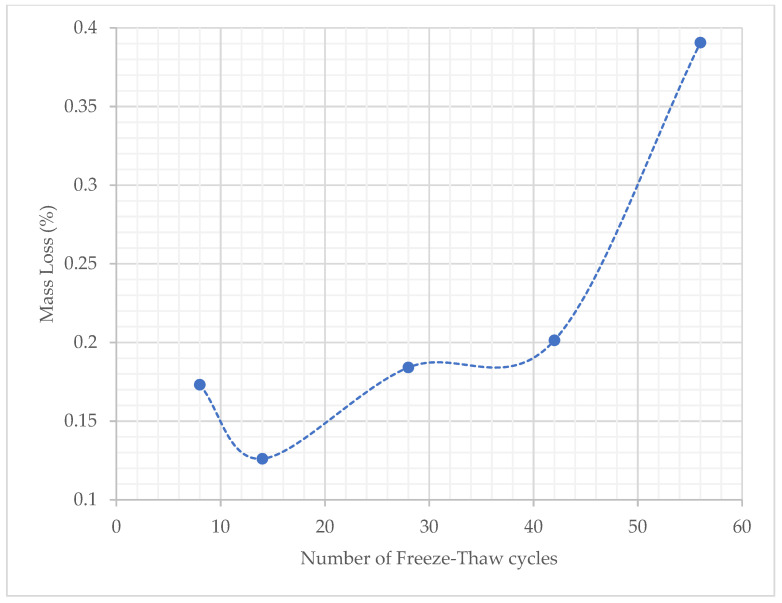
Mass loss (%) vs. Number of Freeze–Thaw cycles.

Standard cube specimens were assessed for freeze–thaw durability as prescribed by CEN/TS 12390-9:2016 [33]. The mass loss due to scaling was recorded after each freeze–thaw cycle and the mass loss due to scaling vs. the number of freeze–thaw cycles the set underwent are provided in Figure 11. The average scaling level at 56 cycles was calculated at 1.56% by mass, a value that is lower than the relative scaling level threshold of 3% by mass, as indicated in the CEN/TS 12390-09 [33] standard. In addition, the total mass of scaled materials at 56 freeze–thaw cycles, over the specimen surface area was also calculated and determined to be 0.98 kg/m^2^, a value just under the threshold value of 1 kg/m^2^ defined by the slab-test method in CEN/TS 12390-09 [33] based on the Swedish Standard SS 13 72 44 [40]. Due to a higher absorption rate initially, as well as the external surface roughness of the specimens, mass loss due to scaling is greater after the first 8 cycles than after 14 cycles. Mass loss due to scaling continues to rise after that, as expected with subsequent freeze–thaw cycles.

The freeze–thaw durability test results indicate that the SFRRC mix with 60% by volume of mineral aggregate replaced by rubber can withstand cycles of extreme temperatures successfully, with minor mass loss due to scaling.

## 4. Numerical Study on the Efficiency of SFRRC as a Strengthening Material

### 4.1. SFRCC Numerical Modeling

**Figure 12:**
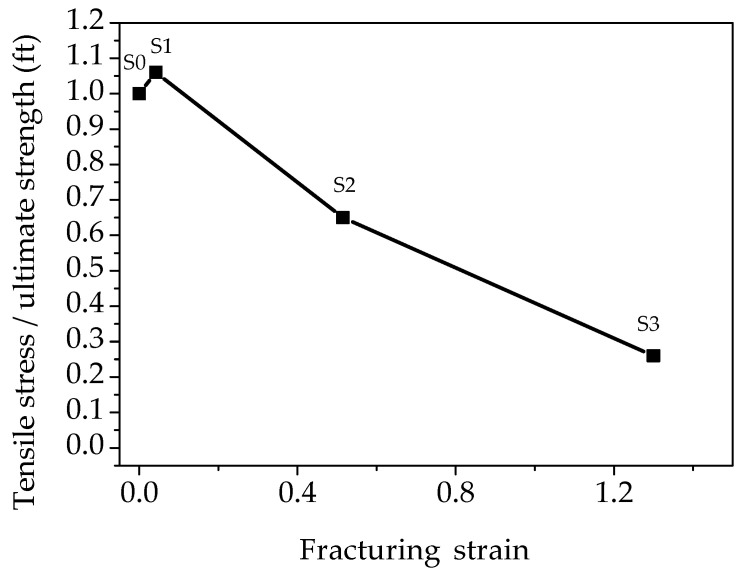
Constitutive model for the tensile stress–strain model of SFRCC. Adapted from [9].

Numerical simulations were conducted using Finite Element Analysis (FEA) software ATENA [41]. For the simulation of SFRCC performance, the compressive strength and modulus of elasticity obtained experimentally (Section 3.1.1 and Section 3.2, respectively), were used. More specifically compressive strength equal to 8.2 MPa and modulus of elasticity equal to 32.6 GPa were used. Regarding the behavior in tension, a previously developed constitutive model for UHPFRC [9] was adopted and inverse analysis was conducted to calculate the characteristic points of the model as presented in Figure 12. From the beginning of the loading until the end of the linear point (S0) there is the uncracked stage, where there is a linear stress–strain behavior. The crack formation takes places after the elastic point (S0), when the crack initiates and is followed by stain hardening until S1 (Figure 12), due to the bridging effect of the fibers. After S1 (Figure 12), the ultimate strength is reached and is followed by decreasing stresses (S2 and S3), and finally there is a complete release of stresses when the crack opens without any stress contribution.

**Figure 13:**
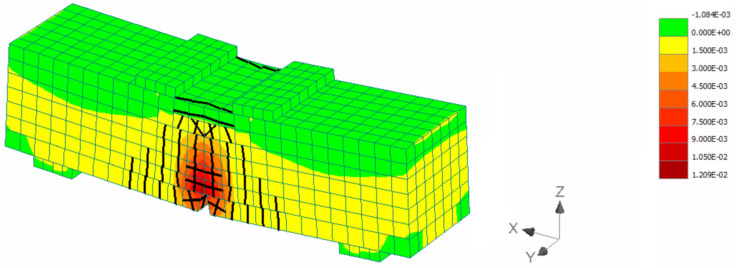
Indicative strain distribution at the maximum load.

For the inverse analysis, the flexural tests results presented in Section 3.1.2 were used. Numerical models were developed to simulate the response of the prisms and analyses were conducted using the model presented in Figure 13 considering different tensile strength values (0.4, 0.6, 0.8, 1.0 MPa). Indicative strain distribution at the maximum load stage for the prism with 0.8 MPa tensile strength is presented in Figure 13. The black lines indicate the crack development while the strain contours represent the strain distribution along the prism span. The maximum compressive strain at the maximum load stage was found at the top of the prism and it was equal to 1%, while the respective maximum tensile strain at the bottom of the prism was 12%.

**Figure 14:**
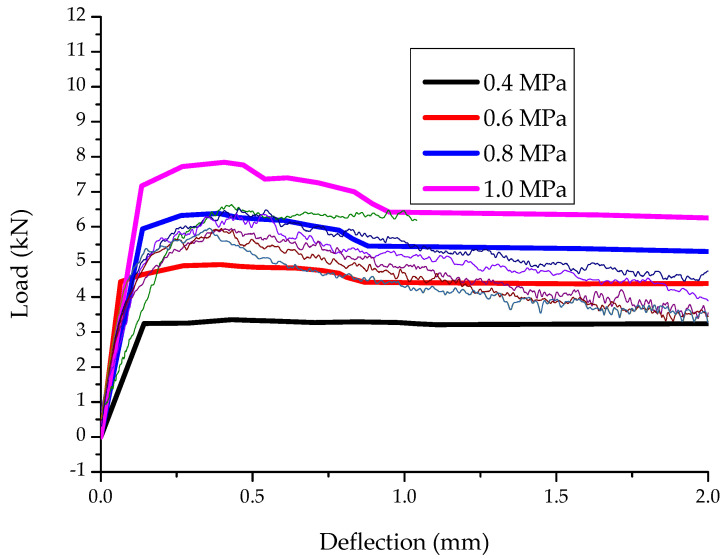
Experimental vs. numerical results of the examined prisms.

The load deflection results of the numerical analyses are compared to the experimental and the results are illustrated in Figure 14. The thinner, colored lines resemble the experimental load deflection data obtained per specimen loaded under bending.

The results of Figure 14 show that a value of 0.8 MPa ultimate tensile strength and the constitutive model of Figure 12 can be used to accurately simulate the behavior of SFRRC. This model was used to evaluate the effectiveness of SFRRC as strengthening material.

### 4.2. Strengthening of Reinforced Concrete Beams Using SFRRC Layer

**Figure 15:**
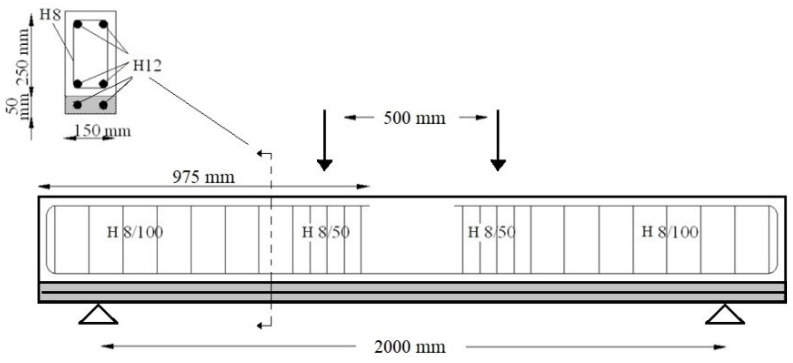
Geometry and reinforcement details of the strengthened beams. Adapted from [42].

Reinforced Concrete beams have been examined to evaluate the efficiency of the use of SFRRC as a strengthening material. The examined specimens are based on a previous experimental study [42] where the performance of large-scale beams strengthened with additional concrete layers was studied. The cross section of the initial beam was 150 mm by 250 mm, the thickness of the additional layer was 50 mm and the total length of the examined specimens was equal to 2200 mm while the span length was 2000 mm. The cylinder compressive strength of the concrete of the initial beam was equal to 39.5 MPa while the respective strength value for the additional reinforced concrete layer was 45.4 MPa. B500 steel reinforcement was used in both the initial beam and the additional layer, and the reinforcement details are illustrated in Figure 15.

**Figure 16:**
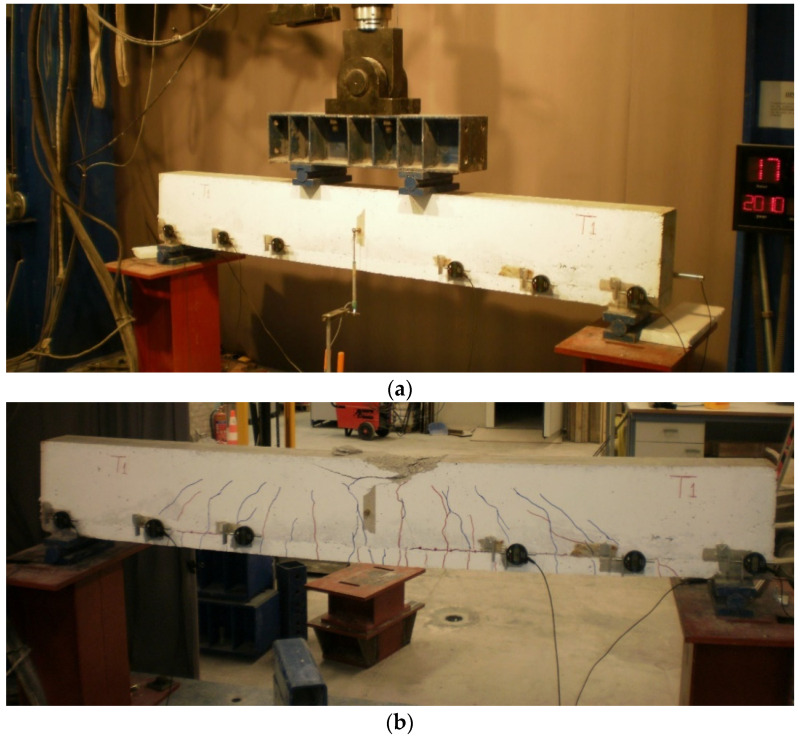
(**a**) Test setup and (**b**) failure mode of the beam strengthened with conventional reinforced concrete layer.

The experimental setup and the failure pattern of one of the strengthened beams with conventional reinforced concrete layer are presented in Figure 16a,b.

Numerical models were also developed for the initial and strengthened beams and the reliability of the models when conventional concrete was used was validated using the experimental results [9].

The same assumptions were used in this study for the simulation of the initial beams. For the strengthening layer, the assumptions presented in Section 4.1 were used with a tensile strength of 0.8 MPa, which was found to be the most appropriate value. Regarding the connection between the strengthening layer and the initial beam, contact elements with coefficient of friction 1.5 and cohesion 1.9 MPa were used, while a shrinkage strain equal to 565 microstrains was also applied to the new concrete layer [9].

**Figure 17:**
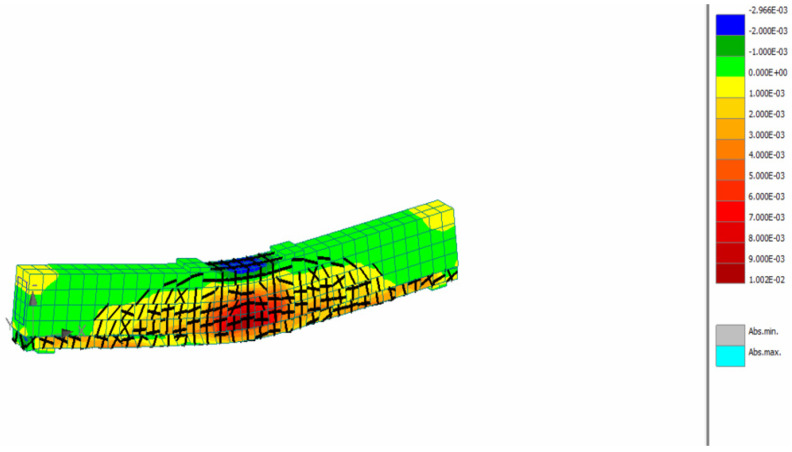
Crack and strain distribution of the SFRRC strengthened beam.

The strain and crack distribution at the SFRRC strengthened beam at the maximum load stage are presented in Figure 17.

**Figure 18:**
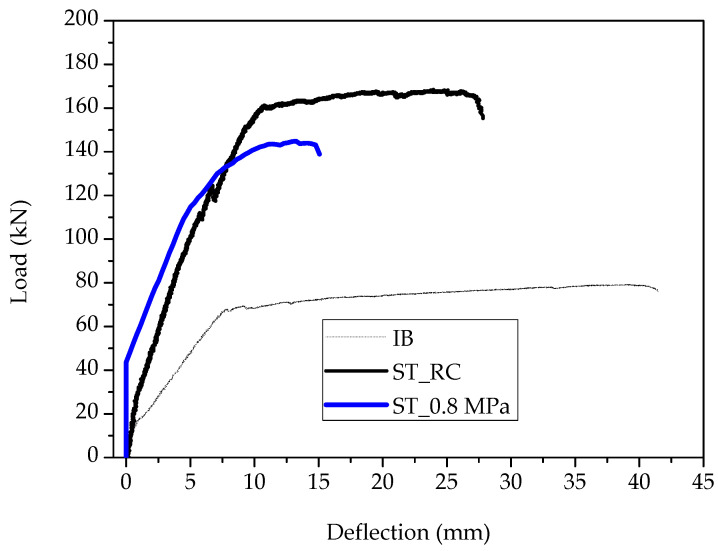
Load-mid span deflection results.

The load mid-span deflection of the SFRRC strengthened beam (ST_0.8 MPa) is compared with the respective experimental results of the beam strengthened with conventional reinforced concrete layer (ST_RC) and the initial beam prior to strengthening (IB) (Figure 18).

The results of Figure 18 indicate that the SFRRC layer can be effectively used to enhance the structural performance of reinforced concrete structural elements since the application of SFRRC led to an 83% increment of the ultimate load as compared to the initial prior-to-strengthening beam. This is quite close to the performance of the strengthened element with conventional reinforced concrete layer (ST_RC), which is attributed to the fact that the main flexural enhancement is due to the presence of steel bars in the strengthening layer. The examined application has a great potential since the use of additional high-damping SFRRC could be effective for the improvement of energy dissipation in addition to the enhancement of ultimate load capacity of existing structures.

## 5. Conclusions

SFRRC that includes a high content of rubber and steel fibers obtained from End-of-life tires is a promising material for sustaining cyclic loading and attains an exceptional strain capacity. Not only does it provide such unique response to loading, but SFRRC that incorporates such high amounts of recycled steel fibers and tire particles obtained from End-of-life tires has a significant environmental value and supports the green deal initiative by promoting a circular economy.

Despite its low compressive strength, SFRRC attains the capacity to withstand long-lasting cyclic loads with significant energy absorption, as indicated by the area under each cycle. The significant energy absorbing capability of the material is also evident from the high damping value computed using the cyclic frequency ω and the mass of the specimen. It was shown that a 60% by volume aggregate replacement by rubber particles in combination with steel fiber reinforcement by recycled steel fibers from End-of-life tires led to improving the damping ratio of the material by 75.4%. Higher damping can lead to the absorption of large force energies and thus constitutes this material appropriate for sustaining impact and earthquake loading. Moreover, the SFRRC mix tested in this study has shown to be durable in freeze–thaw cycles and corrosive environments.

Most importantly, SFRRC is not only an environmentally friendly material that can absorb large volumes of waste (and specifically End-of-life tire products) but has also been proven capable of enhancing the structural performance of reinforced concrete structural elements, as indicated by the results obtained through numerical assessment and comparison to conventional methods. In addition to increasing the load capacity of existing RC structural elements, the high-damping capabilities of SFRRC can enhance the energy dissipation of existing structures. Future studies in this field should be focused on the performance of full structures retrofitted with additional SFRRC elements while the application of SFRRC seismic isolation foundation systems could also be explored. While currently there is a lack of previous research in evaluating SFRRC as a strengthening material for existing RC members, additional research in this area could lead to enabling the use of such novel materials in structural engineering applications.

## Figures and Tables

**Table 1:** Steel Fiber Reinforced Rubberized Concrete with High Rubber Content and Plain Concrete–Mix Design Details.

Mix Constituent	SFRRC Mix Amount (kg/m^3^)	Plain Concrete Mix Amount (kg/m^3^)
Cement CEM I 52,5 N	400.00	400.00
Silica Fume (micro-silica)	100.00	100.00
Fine Mineral Aggregate	310.50	573.70
Coarse Mineral Aggregate	378.00	1147.50
Fine Rubber Particles	169.70	0.00
Coarse Rubber Particles	207.00	0.00
Recycled Tire Steel Fibers	25.00	0.00
Water	225.00	225.00
Superplasticizer	3.61	3.61

**Table 2:** SFRRC Static loading under Compression Results per Experimental Procedure.

Experimental Procedure	Average Value	Variance
Cylinder Compressive Strength	3.499 MPa	0.274
Cube Compressive Strength	8.201 MPa	0.388
Cylinder Modulus of Elasticity	3.263 GPa	0.408

**Table 3:** Average Residual Flexural Strength Values and Coefficients of Variation per CMOD.

	Average Flexural Residual Strength f_Ri_ (MPa)	CoV of Flexural Residual Strength f_Ri_ (MPa)	CMOD (mm)
f_R1_	2.57	0.08	0.5
f_R2_	2.05	0.17	1.5
f_R3_	1.78	0.19	2.5
f_R4_	1.45	0.25	3.5

**Table 4:** SFRRC Impact specimens Damping Ratio, ζ and Frequency, f, Average and Variance.

Damping Ratio, ζ		Frequency, f (Hz)	
Average	Variance	Average	Variance
0.118	0.243	81.00	0.096

**Table 5:** Average Mass Loss, Coefficients of Variation per Chloride Corrosion Test Specimen Set.

Cycle Duration (Days)	NaCl Solution % Concentration	Average Mass Loss (%)	Coefficient of Variation
56 (28 in/28 out)	3	0.98	0.067
112 (56 in/56 out)	3	1.29	0.026
168 (84 in/84 out)	No NaCl	1.23	0.043
168 (84 in/84 out)	3	1.04	0.085

## Data Availability

Data presented in this study are available upon request.
